# Judicial Opinion 131

**DOI:** 10.1099/ijsem.0.006692

**Published:** 2025-02-27

**Authors:** David R. Arahal, Carolee T. Bull, Henrik Christensen, Christopher Dunlap, Maria del Carmen Montero-Calasanz, Charles T. Parker, Peter Vandamme, Antonio Ventosa, Stefano Ventura, Peter Young, Markus Göker

**Affiliations:** 1 Departamento de Microbiología y Ecología, Universitat de València, Valencia, Spain; 2 Department of Plant Pathology and Environmental Microbiology, Pennsylvania State University, 211 Buckhout Lab, University Park, PA 16802, USA; 3 Department of Veterinary and Animal Sciences, University of Copenhagen, 1870 Stigbøjlen 4, Frederiksberg C, Denmark; 4 Crop Bioprotection Research Unit, USDA/ARS/NCAUR, 1815 N. University St, 61604 Peoria, IL, USA; 5 IFAPA Las Torres – Andalusian Institute of Agricultural and Fisheries Research and Training, Cra. Sevilla-Cazalla de la Sierra, 41200, Alcalá del Río, Sevilla, Spain; 6 Department of Energy, Joint Genome Institute, Berkeley, CA 94720, USA; 7 Laboratorium voor Microbiologie, Universiteit Gent, K. L. Ledeganckstraat 35, 9000 Gent, Belgium; 8 Department of Microbiology and Parasitology, Faculty of Pharmacy, University of Sevilla, Sevilla, C/. Prof. Garcia Gonzalez 2, ES-41012 Sevilla, Spain; 9 IRET-CNR, Research Institute on Terrestrial Ecosystems, National Research Council, Via Madonna del Piano 10, 50019 Sesto Fiorentino, Italy; 10 NBCF, National Biodiversity Future Center, Palermo, Italy; 11 Department of Biology, University of York, York YO10 5DD, UK; 12 Leibniz Institute DSMZ – German Collection of Microorganisms and Cell Cultures, Inhoffenstrasse 7B, D-38124 Braunschweig, Germany

**Keywords:** amphibians, bacteria, medical microbiology, nomenclature, protozoa, taxonomy

## Abstract

Opinion 131 addresses a Request for an Opinion asking the Judicial Commission to conserve the genus name *Proteus* Hauser 1885 (Approved Lists 1980) over its earlier homonym, the protozoan genus name *Proteus* Müller 1786. The Judicial Commission agrees that the later homonym is illegitimate and that the replacement of the prokaryotic name *Proteus* would be undesirable. It is also concluded that *Proteus* Müller 1786 is an objectively invalid name under the *International Code of Zoological Nomenclature*. Judicial Opinions 9, 12 and 130 serve as precedents for the conservation of *Proteus* Hauser 1885 (Approved Lists 1980) over *Proteus* Müller 1786. This action is taken here and makes the prokaryotic name *Proteus* legitimate.

## Introduction

The Request for an Opinion by Li [1] concludes that the genus name *Proteus* Hauser 1885 (Approved Lists 1980) [2,3] is illegitimate because it is a later homonym of *Proteus* Müller 1786, the name of a genus of protozoa. This would also make all validly published names of species and subspecies placed within *Proteus* Hauser 1885 (Approved Lists 1980) illegitimate [2,23]. Li [1] considers this undesirable and therefore asks the Judicial Commission to conserve *Proteus* Hauser 1885 (Approved Lists 1980) over *Proteus* Müller 1786.

The action of conserving one name over another, even if the latter is the name of a eukaryote, has only recently been reviewed by the Judicial Commission [24,25]. The present Judicial Opinion can therefore be kept brief and follows the logic of the argumentation set out in the guidelines published by the Commission and in the previous Opinion [24,25]. In what follows, it will first be clarified whether *Proteus* Hauser 1885 (Approved Lists 1980) [2,3] is illegitimate and what the consequences might be. It will then be considered whether, if it is illegitimate, the name could be made legitimate and, if so, whether the Judicial Commission should take such a step.

## Is *Proteus* Hauser 1885 (Approved Lists 1980) illegitimate?

The potential illegitimacy of *Proteus* Hauser 1885 (Approved Lists 1980) [2,3] has already been noted [26,27]. A comprehensive treatment was given by Becker [26], who recognized the two earlier homonyms:

*‘Proteus* Laurenti 1768 (38, p. 35). Type species. *Proteus anguinus* Laurenti 1768. The species described is placed in *Amphibia*. The name is apparently currently accepted and used, as by Noble 1931 (39, p. 483). *Proteus* Mueller 1786 (40, p. 91). Type species. *Proteus diffluens* Mueller 1786. This protozoan generic name was apparently a later homonym of *Proteus* Laurenti 1768’.

Like Li [1], Becker [26] realized that *Proteus* Müller 1786 is itself a later homonym of *Proteus* Laurenti 1768. The relevant code for both names is the *International Code of Zoological Nomenclature* (ICZN) [28]. In the ICZN, the term ‘available name’ roughly corresponds to the term ‘validly published name’ used in the *International Code of Nomenclature of Prokaryotes* (ICNP) [29], while the ICZN terms ‘objectively invalid name’, ‘potentially valid name’ and ‘valid name’ roughly correspond to the ICNP terms ‘illegitimate name’, ‘legitimate name’ and ‘correct name’, respectively. According to Article 52.2 of the ICZN, only the senior homonym can be a valid name; according to Article 60.1, the junior homonym requires a substitute name [28].

As noted by Li [1], Rule 51b(4) of the ICNP is relevant to the legitimacy, or lack thereof, of *Proteus* Hauser 1885 (Approved Lists 1980) [2,3]. It gives as one of the reasons for the illegitimacy of a validly published name:

‘If a new name or combination validly published before 31 December 2000 is a later homonym of a name of a taxon of prokaryotes, fungi, algae, protozoa or viruses’.

Unlike Rule 51b(5), this does not require the earlier homonym to have a particular status or recognition under a particular code. However, Rule 51b(4) limits the earlier homonyms of relevance to viruses and certain groups of eukaryotes [30]. For this reason, the presence of the name *Proteus* Laurenti 1768, a potentially valid name under the ICZN, does not render *Proteus* Hauser 1885 (Approved Lists 1980) illegitimate, but the presence of *Proteus* Müller 1786 does, although the latter is an objectively invalid name under the ICZN.

## Could *Proteus* Hauser 1885 (Approved Lists 1980) be made legitimate?

The power of the Judicial Commission to conserve names over other names is well known [24] and based on Rules 23a and 56b of the ICNP [29]. This extends to the conservation of a prokaryotic name over the name of a eukaryote. Judicial Opinion 130 [25] has reviewed such cases and reiterated that such an action can make an illegitimate name legitimate. Together with Judicial Opinions 9 and 12 [31], Judicial Opinion 130 can also serve as a precedent for the conservation of a prokaryotic name over the name of a eukaryote itself.

## Should *Proteus* Hauser 1885 (Approved Lists 1980) be made legitimate?

Judicial Opinion 130 [25] states that a name should be conserved over an earlier homonym to overcome the illegitimacy of the later homonym if the Judicial Commission has the power to do so and if the later homonym is ‘significantly more important’ than the earlier homonym. Since *Proteus* Müller 1786 cannot even be the valid name of an organism whose naming is regulated by the ICZN [1,26, 28], the Judicial Commission has no doubt that *Proteus* Hauser 1885 (Approved Lists 1980) is the significantly more important name [2,23]. Additional reasons why replacing *Proteus* Hauser 1885 (Approved Lists 1980) would be undesirable are given by Li [1], including the medical importance of some of the species included in *Proteus* Hauser 1885 (Approved Lists 1980).

With regard to *Proteus* Laurenti 1768, it is not only important that this name has no bearing on the legitimacy of *Proteus* Hauser 1885 (Approved Lists 1980), but it is also noteworthy that Article 52.7 of the ICZN implies that *Proteus* Hauser 1885 (Approved Lists 1980) ‘is not a homonym for the purposes of zoological nomenclature’ [28]. The ICZN does not reciprocate Principle 2 of the ICNP [29].

## Conclusion

Principle 1 of the ICNP sets out the ‘four essential points of nomenclature’ [29]. A closer look at these four points reveals that they are in fact opposing forces that need to be balanced against each other in practice [24]. For example, Principle 1(1) and Principle 1(4) can easily conflict, as ‘freedom of taxonomic thought or action’ often results in names that are intended to replace other names [32]. Principle 1(2) and Principle 1(1), considered in isolation, may also lead to different outcomes, since the rejection of names that ‘cause error or confusion’ reduces the ‘stability of names’.

Of the ‘four essential points’, the illegitimacy of a later homonym and its possible replacement are based on Principle 1(2) and are mainly implemented by Section 8 of the ICNP [29]. However, the replacement of an illegitimate name, although provided for in Rule 54, is contrary to Principle 1(1). The conservation of an illegitimate name over an earlier homonym under Rules 23a and 56b is instead a way of implementing Principle 1(1). As in other situations, how best to balance the ‘four essential points’ in the case of *Proteus* must be based on the specifics of the situation.

The negative consequences of the current illegitimacy of *Proteus* Hauser 1885 (Approved Lists 1980) [2,3] are obvious, while those of conserving this name over *Proteus* Müller 1786 are unknown. As long as *Proteus* Hauser 1885 (Approved Lists 1980) is illegitimate, this name and the names of the species in this genus are not available for use under the ICNP and would need to be replaced [29]. For this reason, the Judicial Commission decided to conserve *Proteus* Hauser 1885 (Approved Lists 1980) over *Proteus* Müller 1786. In the first vote, 11 commissioners agreed that *Proteus* Hauser 1885 (Approved Lists 1980) was illegitimate, none voted against and none abstained. Eleven commissioners then voted in favour of the conservation of the name, none voted against and none abstained. One commissioner did not participate in the ballot. (It was also asked whether the name should be rejected if it was not conserved. Two commissioners agreed, nine voted against and none abstained.) The Judicial Commission wishes to reiterate that conservation can, in principle, be applied to any other case of inconvenient homonymy.

## Abbreviations

ICNP: *International Code of Nomenclature of Prokaryotes*
ICZN: *International Code of Zoological Nomenclature*
